# Supplementary material for: Interventional neurorehabilitation for promoting functional recovery post-craniotomy: a proof-of-concept
Source: Sci Rep. 2022 Feb 23;12:3039. doi: 10.1038/s41598-022-06766-8 (PMC8866464; doi:10.1038/s41598-022-06766-8)
Supplement: Supplementary file 1 — Supplementary Information. [file 41598_2022_6766_MOESM1_ESM.docx]

**Supplemental Digital Content:** This video expands on the TMS technique described in the Methods. The video illustrates patient set-up, patient registration, measuring motor threshold, and TMS treatment. The participants consented to publication of his/her image.

<https://drive.google.com/drive/folders/1k_ST-gu1scstgzzy7q0lzSqxEybHghX5?usp=sharing>
